# 2,2′-(Decane-1,10-di­yl)dibenz­imid­azo­lium dichloride trihydrate

**DOI:** 10.1107/S160053680800617X

**Published:** 2008-03-12

**Authors:** Jun-Ming Yi, Yun-Qian Zhang, Sai-Feng Xue, Qian-Jiang Zhu

**Affiliations:** a Center for Research & Development of Fine Chemicals Guizhou University, Guiyang 550025, People’s Republic of China; b Key Laboratory of Macrocyclic and Supramolecular Chemistry of Guizhou Province, Guizhou University, Guiyang 550025, People’s Republic of China; c Institute of Applied Chemistry Guizhou University, Guiyang 550025, People’s Republic of China

## Abstract

The organic cation in the title compound, C_24_H_32_N_4_^2+^·2Cl^−^·3H_2_O, is situated on an inversion centre. The cations, anions and water mol­ecules are linked *via* N—H⋯O, N—H⋯Cl, O—H⋯O and O—H⋯Cl hydrogen bonds and C—H⋯π interactions, forming a three-dimensional framework.

## Related literature

For general background, see: Day & Arnold (2000 ▶); Day *et al.* (2002 ▶); Freeman *et al.* (1981 ▶); Kim *et al.* (2000 ▶); Wang & Joullie (1957 ▶).
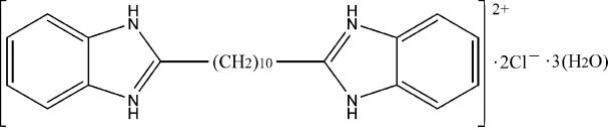


## Experimental

### 

#### Crystal data

C_24_H_32_N_4_^2+^·2Cl^−^·3H_2_O
*M* *_r_* = 501.48
Triclinic, 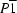

*a* = 10.8482 (6) Å
*b* = 11.5089 (6) Å
*c* = 11.9503 (6) Å
α = 77.619 (2)°
β = 71.501 (2)°
γ = 76.030 (2)°
*V* = 1357.58 (13) Å^3^
*Z* = 2
Mo *K*α radiation
μ = 0.27 mm^−1^
*T* = 293 (2) K
0.29 × 0.24 × 0.16 mm

#### Data collection

Bruker APEXII CCD area-detector diffractometer
Absorption correction: multi-scan (*SADABS*; Bruker, 2005 ▶) *T*_min_ = 0.926, *T*_max_ = 0.958
13250 measured reflections
4702 independent reflections
3802 reflections with *I* > 2σ(*I*)
*R*_int_ = 0.024

#### Refinement

*R*[*F* ^2^ > 2σ(*F* ^2^)] = 0.035
*wR*(*F* ^2^) = 0.089
*S* = 1.06
4702 reflections
298 parameters
H-atom parameters constrained
Δρ_max_ = 0.22 e Å^−3^
Δρ_min_ = −0.18 e Å^−3^

### 

Data collection: *APEX2* (Bruker, 2005 ▶); cell refinement: *SAINT* (Bruker, 2005 ▶); data reduction: *SAINT*; program(s) used to solve structure: *SHELXS97* (Sheldrick, 2008 ▶); program(s) used to refine structure: *SHELXL97* (Sheldrick, 2008 ▶); molecular graphics: *ORTEP-3* (Farrugia, 1997 ▶); software used to prepare material for publication: *WinGX* (Farrugia, 1999 ▶).

## Supplementary Material

Crystal structure: contains datablocks global, I. DOI: 10.1107/S160053680800617X/rk2079sup1.cif

Structure factors: contains datablocks I. DOI: 10.1107/S160053680800617X/rk2079Isup2.hkl

Additional supplementary materials:  crystallographic information; 3D view; checkCIF report

## Figures and Tables

**Table 1:** Hydrogen-bond geometry (Å, °)

*D*—H⋯*A*	*D*—H	H⋯*A*	*D*⋯*A*	*D*—H⋯*A*
N1—H1⋯O3*W*	0.86	1.88	2.7142 (19)	162
N2—H2*A*⋯O2*W*	0.86	1.94	2.7500 (19)	157
N3—H3*A*⋯O1*W*^i^	0.86	1.88	2.7322 (18)	173
N4—H4*A*⋯Cl2	0.86	2.25	3.0823 (15)	163
O1*W*—H1*WA*⋯Cl1^ii^	0.87	2.25	3.1027 (13)	168
O1*W*—H1*WB*⋯Cl1^iii^	0.89	2.21	3.0804 (13)	166
O2*W*—H2*WA*⋯O1*W*	0.97	1.96	2.8763 (18)	158
O2*W*—H2*WB*⋯Cl2^iv^	0.90	2.28	3.1703 (13)	170
O3*W*—H3*WB*⋯Cl1	0.93	2.20	3.0912 (13)	162
O3*W*—H3*WA*⋯Cl2	0.92	2.21	3.1229 (13)	172
C11—H11*B*⋯*Cg*2	0.97	3.17	3.847 (3)	128
C22—H22*A*⋯*Cg*1^v^	0.97	2.92	3.863 (3)	165

Symmetry codes: (i) 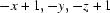; (ii) 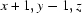; (iii) 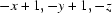; (iv) 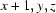; (v) 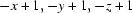. *Cg1*, *Cg2* are the centroids of the C1–C6 and C13–C18 benzene rings, respectively.

**Fig. 1.**
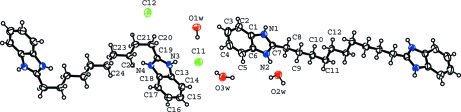
The molecular structure of I with the atom numbering scheme. Displacement ellipsoids are drawn at 50% probability level. H atoms are presented as a small spheres of arbitrary radius.

## supplementary crystallographic information

### Comment

We prepared and present a new 'axle' polyaromatic compound (I) containing multiple functional groups that can develop strong intermolecular interactions with cucurbit[*n*]urils (*CB*[*n*]) (Freeman *et al.*, 1981; Day & Arnold, 2000; Day *et al.*, 2002; Kim *et al.*, 2000).

The structure of I, [C_24_H_32_N_4_]^2+^.2Cl^-^.3(H_2_O), contains two independent molecules, which occupy the center of symmetry positions in the middle of C12–C12^i^ and C24–C24^ii^ bonds, respectively (symmetry codes: (i) -*x* + 2, -*y* + 3, -*z*, (ii) -*x*, -*y*, -*z* + 2). The angle between the plane of the phenyl rings and the plane through C10, C11, C12, C12^i^, C11^i^, C10^i^ chain is 86.74 (9) Å, and the plane through C22, C23, C24, C24^ii^, C23^ii^, C22^ii^ chain is 89.26 (10) Å. The cations, anions and water molecules are linked *via* N–H···O, N–H···Cl, O–H···O, O–H···Cl hydrogen bonds and C—H···π intreactions forming three–dimensional framework (see table, *Cg1*, *Cg2* are the centroid of the C1/C6–benzene ring and C13/C18–benzene ring, respectively).

### Experimental

A solution of *o*–phenylenedimine (5.40 g, 0.05 mol) and 1,10–decanedicarboxylic acid (5.80 g, 0.025 mol) were reflux for 12 h in 70 ml of 4*M* HCl, the reaction mixture was cooled for one day and the crystals of I was removed by filtration and dried. The crystals of the title compound suitable for *X*–ray diffraction were obtained by dissolving in water and standing at room temperature after several days (Wang & Joullie, 1957). Yield: 25%.

### Refinement

Water H atoms were located in a difference Fourier synthesis and refined in their as–found positions relative to O atoms with *U*_iso_(H) = 1.2*U*_eq_(O). All other H atoms were placed in calculated positions and refined as riding, with C—H = 0.93–0.97 Å, N—H = 0.86 Å and with *U*_iso_(H) = 1.2*U*_eq_(C, N).

### Crystal data

**Table:**

C_24_H_32_N_4_^2+^·2Cl^–^·3H_2_O	*Z* = 2
*M**_r_* = 501.48	*F*_000_ = 536
Triclinic, *P*1	*D*_x_ = 1.227 Mg m^−^^3^
Hall symbol: -P 1	Mo *K*α radiation λ = 0.71073 Å
*a* = 10.8482 (6) Å	Cell parameters from 13250 reflections
*b* = 11.5089 (6) Å	θ = 1.8–25.0º
*c* = 11.9503 (6) Å	µ = 0.27 mm^−^^1^
α = 77.619 (2)º	*T* = 293 (2) K
β = 71.501 (2)º	Prism, colourless
γ = 76.030 (2)º	0.29 × 0.24 × 0.16 mm
*V* = 1357.58 (13) Å^3^	

### Data collection

**Table:**

Bruker APEXII CCD area-detector diffractometer	4702 independent reflections
Radiation source: Fine–focus sealed tube	3802 reflections with *I* > 2σ(*I*)
Monochromator: Graphite	*R*_int_ = 0.024
*T* = 293(2) K	θ_max_ = 25.0º
φ and ω scans	θ_min_ = 1.8º
Absorption correction: multi-scan(SADABS; Bruker, 2005)	*h* = −12→12
*T*_min_ = 0.926, *T*_max_ = 0.958	*k* = −12→13
13250 measured reflections	*l* = −11→14

### Refinement

**Table:**

Refinement on *F*^2^	Secondary atom site location: Difmap
Least-squares matrix: Full	Hydrogen site location: Geom
*R*[*F*^2^ > 2σ(*F*^2^)] = 0.035	H-atom parameters constrained
*wR*(*F*^2^) = 0.089	*w* = 1/[σ^2^(*F*_o_^2^) + (0.0377*P*)^2^ + 0.299*P*] where *P* = (*F*_o_^2^ + 2*F*_c_^2^)/3
*S* = 1.06	(Δ/σ)_max_ < 0.001
4702 reflections	Δρ_max_ = 0.22 e Å^−^^3^
298 parameters	Δρ_min_ = −0.18 e Å^−^^3^
Primary atom site location: Direct	Extinction correction: none

### Special details

**Table:**

Geometry. All s.u.'s (except the s.u. in the dihedral angle between two l.s. planes) are estimated using the full covariance matrix. The cell s.u.'s are taken into account individually in the estimation of s.u.'s in distances, angles and torsion angles; correlations between s.u.'s in cell parameters are only used when they are defined by crystal symmetry. An approximate (isotropic) treatment of cell s.u.'s is used for estimating s.u.'s involving l.s. planes.
Refinement. Refinement of *F*^2^ against ALL reflections. The weighted *R*–factor w*R* and goodness of fit *S* are based on *F*^2^, conventional *R*–factors *R* are based on *F*, with *F* set to zero for negative *F*^2^. The threshold expression of *F*^2^ > 2σ(*F*^2^) is used only for calculating *R*–factors(gt) *etc*. and is not relevant to the choice of reflections for refinement. *R*–factors based on *F*^2^ are statistically about twice as large as those based on *F*, and *RR*–factors based on ALL data will be even larger.

### Fractional atomic coordinates and isotropic or equivalent isotropic displacement parameters (Å^2^)

**Table:**

	*x*	*y*	*z*	*U*_iso_*/*U*_eq_	
C1	0.64008 (18)	0.65504 (16)	0.11143 (15)	0.0407 (4)	
C2	0.6254 (2)	0.78026 (18)	0.09401 (19)	0.0572 (5)	
H2	0.5447	0.8308	0.0923	0.069*	
C3	0.7371 (2)	0.8257 (2)	0.07942 (19)	0.0624 (6)	
H3	0.7317	0.9091	0.0668	0.075*	
C4	0.8579 (2)	0.7500 (2)	0.08310 (17)	0.0570 (6)	
H4	0.9307	0.7845	0.0729	0.068*	
C5	0.87257 (19)	0.62624 (19)	0.10132 (16)	0.0487 (5)	
H5	0.9530	0.5759	0.1043	0.058*	
C6	0.76037 (17)	0.58019 (16)	0.11513 (15)	0.0387 (4)	
C7	0.61491 (17)	0.46486 (15)	0.13702 (15)	0.0364 (4)	
C8	0.55400 (18)	0.35944 (16)	0.14626 (17)	0.0418 (4)	
H8A	0.5851	0.3315	0.0693	0.050*	
H8B	0.4589	0.3864	0.1634	0.050*	
C9	0.58230 (18)	0.25312 (15)	0.24031 (15)	0.0392 (4)	
H9A	0.5473	0.2783	0.3186	0.047*	
H9B	0.6772	0.2260	0.2255	0.047*	
C10	0.51925 (18)	0.14950 (16)	0.23773 (16)	0.0409 (4)	
H10A	0.4243	0.1771	0.2548	0.049*	
H10B	0.5514	0.1281	0.1579	0.049*	
C11	0.54728 (18)	0.03713 (16)	0.32589 (16)	0.0415 (4)	
H11A	0.6422	0.0092	0.3085	0.050*	
H11B	0.5093	−0.0263	0.3148	0.050*	
C12	0.49251 (16)	0.05731 (15)	0.45552 (15)	0.0384 (4)	
H12A	0.5374	0.1144	0.4686	0.046*	
H12B	0.3994	0.0935	0.4705	0.046*	
C13	0.16012 (15)	−0.00823 (15)	0.50147 (15)	0.0340 (4)	
C14	0.19499 (17)	−0.13242 (16)	0.50035 (18)	0.0430 (4)	
H14	0.1875	−0.1877	0.5703	0.052*	
C15	0.24119 (18)	−0.16880 (18)	0.38967 (19)	0.0484 (5)	
H15	0.2662	−0.2511	0.3848	0.058*	
C16	0.25169 (17)	−0.08601 (18)	0.28440 (18)	0.0463 (5)	
H16	0.2826	−0.1148	0.2116	0.056*	
C17	0.21752 (16)	0.03697 (17)	0.28543 (16)	0.0405 (4)	
H17	0.2246	0.0921	0.2153	0.049*	
C18	0.17194 (15)	0.07423 (15)	0.39687 (15)	0.0329 (4)	
C19	0.09620 (15)	0.17706 (16)	0.55014 (15)	0.0353 (4)	
C20	0.04445 (17)	0.27781 (17)	0.62225 (17)	0.0454 (5)	
H20A	0.0525	0.2469	0.7023	0.055*	
H20B	−0.0489	0.3056	0.6283	0.055*	
C21	0.11453 (17)	0.38577 (16)	0.57205 (17)	0.0436 (4)	
H21A	0.1086	0.4154	0.4912	0.052*	
H21B	0.0685	0.4502	0.6194	0.052*	
C22	0.25918 (16)	0.35777 (15)	0.57035 (17)	0.0398 (4)	
H22A	0.2659	0.3266	0.6507	0.048*	
H22B	0.3064	0.2952	0.5210	0.048*	
C23	0.32401 (18)	0.46815 (16)	0.52292 (18)	0.0463 (5)	
H23A	0.2725	0.5324	0.5688	0.056*	
H23B	0.3220	0.4959	0.4408	0.056*	
C24	0.46640 (18)	0.44573 (17)	0.52768 (17)	0.0453 (5)	
H24A	0.4678	0.4235	0.6103	0.054*	
H24B	0.5167	0.3778	0.4864	0.054*	
Cl1	0.18425 (5)	0.85742 (4)	0.02569 (4)	0.04826 (14)	
Cl2	0.16011 (5)	0.39667 (4)	0.21933 (4)	0.05033 (15)	
N1	0.55187 (14)	0.57926 (12)	0.12641 (13)	0.0397 (4)	
H1	0.4698	0.6027	0.1285	0.048*	
N2	0.73979 (14)	0.46237 (13)	0.13165 (13)	0.0400 (4)	
H2A	0.7986	0.3979	0.1375	0.048*	
N3	0.11251 (13)	0.06012 (13)	0.59441 (12)	0.0362 (3)	
H3A	0.0962	0.0311	0.6691	0.043*	
N4	0.13153 (13)	0.18871 (12)	0.43127 (12)	0.0355 (3)	
H4A	0.1296	0.2562	0.3835	0.043*	
O1W	0.95820 (12)	0.03649 (11)	0.16974 (10)	0.0452 (3)	
H1WA	1.0290	−0.0060	0.1285	0.054*	
H1WB	0.9039	0.0643	0.1235	0.054*	
O3W	0.28364 (12)	0.62540 (11)	0.18261 (12)	0.0529 (4)	
H3WA	0.2395	0.5627	0.1963	0.063*	
H3WB	0.2443	0.6840	0.1315	0.063*	
O2W	0.92820 (12)	0.28229 (11)	0.20533 (12)	0.0494 (3)	
H2WA	0.9616	0.2021	0.1829	0.059*	
H2WB	0.9970	0.3157	0.1996	0.059*	

### Atomic displacement parameters (Å^2^)

**Table:**

	*U*^11^	*U*^22^	*U*^33^	*U*^12^	*U*^13^	*U*^23^
C1	0.0453 (10)	0.0372 (10)	0.0372 (10)	−0.0113 (8)	−0.0083 (8)	−0.0014 (8)
C2	0.0637 (13)	0.0390 (12)	0.0617 (14)	−0.0091 (10)	−0.0123 (11)	−0.0012 (10)
C3	0.0850 (17)	0.0438 (12)	0.0578 (14)	−0.0296 (12)	−0.0094 (12)	−0.0023 (10)
C4	0.0653 (14)	0.0640 (14)	0.0463 (12)	−0.0362 (12)	−0.0062 (10)	−0.0053 (10)
C5	0.0473 (11)	0.0588 (13)	0.0423 (11)	−0.0208 (10)	−0.0090 (9)	−0.0059 (9)
C6	0.0433 (10)	0.0390 (10)	0.0324 (10)	−0.0116 (8)	−0.0085 (8)	−0.0016 (8)
C7	0.0371 (9)	0.0377 (10)	0.0343 (10)	−0.0082 (8)	−0.0114 (8)	−0.0013 (7)
C8	0.0403 (10)	0.0391 (10)	0.0481 (11)	−0.0069 (8)	−0.0169 (8)	−0.0044 (8)
C9	0.0433 (10)	0.0389 (10)	0.0368 (10)	−0.0119 (8)	−0.0108 (8)	−0.0044 (8)
C10	0.0404 (10)	0.0415 (10)	0.0437 (11)	−0.0131 (8)	−0.0119 (8)	−0.0062 (8)
C11	0.0398 (10)	0.0373 (10)	0.0492 (11)	−0.0105 (8)	−0.0119 (8)	−0.0079 (8)
C12	0.0334 (9)	0.0320 (9)	0.0507 (11)	−0.0080 (8)	−0.0123 (8)	−0.0050 (8)
C13	0.0280 (8)	0.0401 (10)	0.0370 (10)	−0.0090 (7)	−0.0113 (7)	−0.0061 (8)
C14	0.0382 (10)	0.0393 (11)	0.0549 (12)	−0.0098 (8)	−0.0197 (9)	−0.0012 (9)
C15	0.0408 (10)	0.0416 (11)	0.0704 (14)	−0.0061 (9)	−0.0215 (10)	−0.0174 (10)
C16	0.0375 (10)	0.0571 (13)	0.0533 (12)	−0.0097 (9)	−0.0144 (9)	−0.0235 (10)
C17	0.0351 (9)	0.0524 (12)	0.0382 (10)	−0.0133 (8)	−0.0123 (8)	−0.0068 (8)
C18	0.0266 (8)	0.0361 (10)	0.0392 (10)	−0.0091 (7)	−0.0113 (7)	−0.0058 (8)
C19	0.0247 (8)	0.0436 (11)	0.0397 (10)	−0.0099 (7)	−0.0085 (7)	−0.0080 (8)
C20	0.0326 (9)	0.0541 (12)	0.0511 (12)	−0.0083 (8)	−0.0063 (8)	−0.0192 (9)
C21	0.0388 (10)	0.0386 (10)	0.0543 (12)	−0.0002 (8)	−0.0137 (9)	−0.0163 (9)
C22	0.0382 (10)	0.0346 (10)	0.0471 (11)	−0.0055 (8)	−0.0121 (8)	−0.0087 (8)
C23	0.0490 (11)	0.0386 (11)	0.0537 (12)	−0.0111 (9)	−0.0149 (9)	−0.0084 (9)
C24	0.0477 (11)	0.0462 (11)	0.0463 (11)	−0.0153 (9)	−0.0126 (9)	−0.0093 (9)
Cl1	0.0498 (3)	0.0453 (3)	0.0464 (3)	0.0017 (2)	−0.0178 (2)	−0.0055 (2)
Cl2	0.0542 (3)	0.0429 (3)	0.0557 (3)	−0.0152 (2)	−0.0192 (2)	0.0017 (2)
N1	0.0358 (8)	0.0348 (9)	0.0464 (9)	−0.0034 (7)	−0.0143 (7)	−0.0009 (7)
N2	0.0367 (8)	0.0351 (8)	0.0469 (9)	−0.0047 (6)	−0.0129 (7)	−0.0037 (7)
N3	0.0331 (8)	0.0430 (9)	0.0337 (8)	−0.0114 (6)	−0.0105 (6)	−0.0015 (7)
N4	0.0336 (7)	0.0336 (8)	0.0386 (9)	−0.0086 (6)	−0.0103 (6)	−0.0012 (6)
O1W	0.0441 (7)	0.0501 (8)	0.0374 (7)	0.0007 (6)	−0.0125 (6)	−0.0075 (6)
O3W	0.0435 (7)	0.0373 (7)	0.0740 (10)	−0.0072 (6)	−0.0192 (7)	0.0034 (6)
O2W	0.0416 (7)	0.0412 (7)	0.0683 (9)	−0.0058 (6)	−0.0202 (6)	−0.0092 (6)

### Geometric parameters (Å, °)

**Table:**

C1—C6	1.387 (3)	C15—C16	1.397 (3)
C1—C2	1.388 (3)	C15—H15	0.9300
C1—N1	1.391 (2)	C16—C17	1.376 (3)
C2—C3	1.380 (3)	C16—H16	0.9300
C2—H2	0.9300	C17—C18	1.388 (2)
C3—C4	1.396 (3)	C17—H17	0.9300
C3—H3	0.9300	C18—N4	1.391 (2)
C4—C5	1.372 (3)	C19—N3	1.327 (2)
C4—H4	0.9300	C19—N4	1.334 (2)
C5—C6	1.392 (2)	C19—C20	1.486 (2)
C5—H5	0.9300	C20—C21	1.526 (2)
C6—N2	1.390 (2)	C20—H20A	0.9700
C7—N1	1.329 (2)	C20—H20B	0.9700
C7—N2	1.329 (2)	C21—C22	1.518 (2)
C7—C8	1.485 (2)	C21—H21A	0.9700
C8—C9	1.517 (2)	C21—H21B	0.9700
C8—H8A	0.9700	C22—C23	1.516 (2)
C8—H8B	0.9700	C22—H22A	0.9700
C9—C10	1.521 (2)	C22—H22B	0.9700
C9—H9A	0.9700	C23—C24	1.521 (3)
C9—H9B	0.9700	C23—H23A	0.9700
C10—C11	1.518 (2)	C23—H23B	0.9700
C10—H10A	0.9700	C24—C24^ii^	1.520 (3)
C10—H10B	0.9700	C24—H24A	0.9700
C11—C12	1.520 (2)	C24—H24B	0.9700
C11—H11A	0.9700	N1—H1	0.8600
C11—H11B	0.9700	N2—H2A	0.8600
C12—C12^i^	1.517 (3)	N3—H3A	0.8600
C12—H12A	0.9700	N4—H4A	0.8600
C12—H12B	0.9700	O1W—H1WA	0.8680
C13—C18	1.388 (2)	O1W—H1WB	0.8916
C13—C14	1.389 (2)	O3W—H3WA	0.9188
C13—N3	1.390 (2)	O3W—H3WB	0.9275
C14—C15	1.375 (3)	O2W—H2WA	0.9681
C14—H14	0.9300	O2W—H2WB	0.8971
			
C6—C1—C2	121.54 (18)	C16—C15—H15	119.0
C6—C1—N1	106.43 (15)	C17—C16—C15	121.84 (18)
C2—C1—N1	132.02 (18)	C17—C16—H16	119.1
C3—C2—C1	116.3 (2)	C15—C16—H16	119.1
C3—C2—H2	121.8	C16—C17—C18	116.30 (17)
C1—C2—H2	121.8	C16—C17—H17	121.8
C2—C3—C4	121.9 (2)	C18—C17—H17	121.8
C2—C3—H3	119.0	C17—C18—C13	121.77 (16)
C4—C3—H3	119.0	C17—C18—N4	131.94 (16)
C5—C4—C3	121.93 (19)	C13—C18—N4	106.29 (14)
C5—C4—H4	119.0	N3—C19—N4	108.88 (15)
C3—C4—H4	119.0	N3—C19—C20	125.13 (16)
C4—C5—C6	116.28 (19)	N4—C19—C20	125.99 (16)
C4—C5—H5	121.9	C19—C20—C21	114.39 (15)
C6—C5—H5	121.9	C19—C20—H20A	108.7
C1—C6—N2	106.09 (15)	C21—C20—H20A	108.7
C1—C6—C5	121.99 (17)	C19—C20—H20B	108.7
N2—C6—C5	131.91 (17)	C21—C20—H20B	108.7
N1—C7—N2	109.12 (15)	H20A—C20—H20B	107.6
N1—C7—C8	123.98 (15)	C22—C21—C20	114.17 (15)
N2—C7—C8	126.82 (16)	C22—C21—H21A	108.7
C7—C8—C9	115.36 (15)	C20—C21—H21A	108.7
C7—C8—H8A	108.4	C22—C21—H21B	108.7
C9—C8—H8A	108.4	C20—C21—H21B	108.7
C7—C8—H8B	108.4	H21A—C21—H21B	107.6
C9—C8—H8B	108.4	C23—C22—C21	112.45 (15)
H8A—C8—H8B	107.5	C23—C22—H22A	109.1
C8—C9—C10	110.33 (14)	C21—C22—H22A	109.1
C8—C9—H9A	109.6	C23—C22—H22B	109.1
C10—C9—H9A	109.6	C21—C22—H22B	109.1
C8—C9—H9B	109.6	H22A—C22—H22B	107.8
C10—C9—H9B	109.6	C22—C23—C24	114.00 (16)
H9A—C9—H9B	108.1	C22—C23—H23A	108.8
C11—C10—C9	113.90 (14)	C24—C23—H23A	108.8
C11—C10—H10A	108.8	C22—C23—H23B	108.8
C9—C10—H10A	108.8	C24—C23—H23B	108.8
C11—C10—H10B	108.8	H23A—C23—H23B	107.6
C9—C10—H10B	108.8	C24^ii^—C24—C23	113.7 (2)
H10A—C10—H10B	107.7	C24^ii^—C24—H24A	108.8
C10—C11—C12	113.70 (15)	C23—C24—H24A	108.8
C10—C11—H11A	108.8	C24^ii^—C24—H24B	108.8
C12—C11—H11A	108.8	C23—C24—H24B	108.8
C10—C11—H11B	108.8	H24A—C24—H24B	107.7
C12—C11—H11B	108.8	C7—N1—C1	109.03 (14)
H11A—C11—H11B	107.7	C7—N1—H1	125.5
C12^i^—C12—C11	113.96 (18)	C1—N1—H1	125.5
C12^i^—C12—H12A	108.8	C7—N2—C6	109.33 (15)
C11—C12—H12A	108.8	C7—N2—H2A	125.3
C12^i^—C12—H12B	108.8	C6—N2—H2A	125.3
C11—C12—H12B	108.8	C19—N3—C13	109.52 (14)
H12A—C12—H12B	107.7	C19—N3—H3A	125.2
C18—C13—C14	121.89 (16)	C13—N3—H3A	125.2
C18—C13—N3	106.14 (14)	C19—N4—C18	109.17 (14)
C14—C13—N3	131.96 (16)	C19—N4—H4A	125.4
C15—C14—C13	116.09 (17)	C18—N4—H4A	125.4
C15—C14—H14	122.0	H1WA—O1W—H1WB	107.0
C13—C14—H14	122.0	H3WA—O3W—H3WB	103.3
C14—C15—C16	122.10 (18)	H2WA—O2W—H2WB	108.7
C14—C15—H15	119.0		
			
C6—C1—C2—C3	0.7 (3)	N3—C13—C18—C17	179.89 (14)
N1—C1—C2—C3	−178.45 (19)	C14—C13—C18—N4	−178.69 (14)
C1—C2—C3—C4	−0.6 (3)	N3—C13—C18—N4	0.28 (16)
C2—C3—C4—C5	0.1 (3)	N3—C19—C20—C21	140.24 (17)
C3—C4—C5—C6	0.4 (3)	N4—C19—C20—C21	−41.0 (2)
C2—C1—C6—N2	−179.04 (17)	C19—C20—C21—C22	−64.7 (2)
N1—C1—C6—N2	0.27 (19)	C20—C21—C22—C23	−178.49 (16)
C2—C1—C6—C5	−0.2 (3)	C21—C22—C23—C24	176.07 (16)
N1—C1—C6—C5	179.12 (16)	C22—C23—C24—C24^ii^	176.07 (19)
C4—C5—C6—C1	−0.4 (3)	N2—C7—N1—C1	1.0 (2)
C4—C5—C6—N2	178.15 (18)	C8—C7—N1—C1	−175.79 (16)
N1—C7—C8—C9	−136.00 (18)	C6—C1—N1—C7	−0.79 (19)
N2—C7—C8—C9	47.8 (2)	C2—C1—N1—C7	178.4 (2)
C7—C8—C9—C10	−177.80 (16)	N1—C7—N2—C6	−0.9 (2)
C8—C9—C10—C11	177.68 (15)	C8—C7—N2—C6	175.85 (16)
C9—C10—C11—C12	62.6 (2)	C1—C6—N2—C7	0.35 (19)
C10—C11—C12—C12^i^	173.94 (17)	C5—C6—N2—C7	−178.35 (19)
C18—C13—C14—C15	−0.3 (2)	N4—C19—N3—C13	−0.13 (17)
N3—C13—C14—C15	−178.99 (16)	C20—C19—N3—C13	178.79 (15)
C13—C14—C15—C16	−0.4 (3)	C18—C13—N3—C19	−0.10 (17)
C14—C15—C16—C17	0.6 (3)	C14—C13—N3—C19	178.73 (17)
C15—C16—C17—C18	−0.1 (2)	N3—C19—N4—C18	0.31 (17)
C16—C17—C18—C13	−0.7 (2)	C20—C19—N4—C18	−178.60 (15)
C16—C17—C18—N4	178.80 (16)	C17—C18—N4—C19	−179.93 (17)
C14—C13—C18—C17	0.9 (2)	C13—C18—N4—C19	−0.37 (17)

Symmetry codes: (i) −*x*+1, −*y*, −*z*+1; (ii) −*x*+1, −*y*+1, −*z*+1.

### Hydrogen-bond geometry (Å, °)

**Table:**

*D*—H···*A*	*D*—H	H···*A*	*D*···*A*	*D*—H···*A*
N1—H1···O3W	0.86	1.88	2.7142 (19)	162
N2—H2A···O2W	0.86	1.94	2.7500 (19)	157
N3—H3A···O1W^i^	0.86	1.88	2.7322 (18)	173
N4—H4A···Cl2	0.86	2.25	3.0823 (15)	163
O1W—H1WA···Cl1^iii^	0.87	2.25	3.1027 (13)	168
O1W—H1WB···Cl1^iv^	0.89	2.21	3.0804 (13)	166
O2W—H2WA···O1W	0.97	1.96	2.8763 (18)	158
O2W—H2WB···Cl2^v^	0.90	2.28	3.1703 (13)	170
O3W—H3WB···Cl1	0.93	2.20	3.0912 (13)	162
O3W—H3WA···Cl2	0.92	2.21	3.1229 (13)	172
C11—H11B···Cg2	0.97	3.17	3.847 (3)	128
C22—H22A···Cg1^ii^	0.97	2.92	3.863 (3)	165

Symmetry codes: (i) −*x*+1, −*y*, −*z*+1; (iii) *x*+1, *y*−1, *z*; (iv) −*x*+1, −*y*+1, −*z*; (v) *x*+1, *y*, *z*; (ii) −*x*+1, −*y*+1, −*z*+1.
